# Behavioral and social predictors of COVID-19 vaccine uptake among persons with disabilities in Kenya

**DOI:** 10.3389/fpubh.2024.1472677

**Published:** 2024-12-12

**Authors:** Josphat Martin Muchangi, Rogers Moraro, Jarim Omogi, Abrar Alasmari, Sheillah Simiyu, Ana Bolio, Lennah Kanyangi, Rehema Mwema, Rose Bukania, Sarah Kosgei

**Affiliations:** ^1^Department of Population Health and Environment, Amref Health Africa, Nairobi, Kenya; ^2^Department of Community Health, Amref International University, Nairobi, Kenya; ^3^Department of Infectious Disease Epidemiology, London School of Hygiene and Tropical Medicine, London, United Kingdom; ^4^Urbanization and Wellbeing Unit, African Population and Health Research Center, Nairobi, Kenya; ^5^State Department for Social Protection and Senior Citizen Affairs, Ministry of Labour and Social Protection, Government of Kenya, Nairobi, Kenya

**Keywords:** COVID-19, persons with disability, vaccine uptake, behavioral and social predictors, drivers of vaccination

## Abstract

Access and uptake of COVID-19 vaccine by persons with disabilities remains largely unknown in low-and middle-income countries, despite the unique barriers they face, their special vulnerabilities and higher risk to severe outcomes. We aimed to identify behavioral and social predictors of COVID-19 uptake among persons with disability in Kenya. A convergent parallel mixed method study design was conducted among 792 persons with disability in four regions (counties) in Kenya. Purposive sampling was used to identify the respondents from the National Council for Persons with Disabilities Registration database. Quantitative data were analyzed using STATA statistical analysis software (version 14). Chi-square (*X*^2^) and Fisher's exact tests were used to test for differences in categorical variables; and multivariate regression analysis done to ascertain the factors that influence the uptake of COVID-19 among persons with disabilities in Kenya. Qualitative data from 7 Focus Group Discussions and 4 Key Informant Interviews were transcribed and themes developed using the Behavioral and Social Drivers of vaccination framework by the World Health Organization. Approximately 59% of persons with disabilities reported to be fully vaccinated with significant disparities noted among those with cognition (34.2%) and self-care (36.6%) impairments. Key predictors of vaccine uptake included confidence in vaccine benefits (Odds ratio [OR]; 11.3, 95% CI[5.2–24.2]), health worker recommendation (OR; 2.6 [1.8–3.7]), employment (OR; 2.1 [1.4–3.1]), perceived risk (OR; 2.0 [1.3–3.1]), age 18–24 years (OR; 0.18 [0.09–0.36]), and rural area of residence (OR; 0.48 [0.29–0.79]). The primary reasons for low uptake included perceived negative vaccine effects and lack of adequate information. Qualitative findings revealed unique motivations for vaccination among persons with disabilities (PWDs), including safeguarding against risks from assistive devices and the influence of political leaders. Barriers included perceived vaccine effects, transportation challenges, and limited access to trusted information, highlighting the need for targeted sensitization, improved healthcare worker engagement, and collaboration with PWD organizations. Subsequent vaccination deployments should map and reach people in all disability domains through relevant institutions of PWDs and localized vaccination campaigns. Related communication strategies should leverage the credibility and trust in health workers and behavior change techniques that inspire confidence in vaccines to improve vaccine uptake.

## Background

The COVID-19 pandemic exacerbated inequalities embedded in global health systems, disproportionately impacting vulnerable populations, particularly persons with disabilities (PWDs). Throughout the pandemic, PWDs faced heightened risks of infection, severe health outcomes, and profound socioeconomic consequences due to barriers such as limited access to information, communication difficulties, and reduced availability of essential healthcare services (1, 2). Globally, vaccine inequity prolonged the crisis, amplifying the disparity and further marginalizing those at high risk (3). Despite their status as a high-priority group, many governments were slow to implement inclusive COVID-19 response plans that addressed the unique needs of PWDs (4). Research showed that while innovative care approaches and inclusive policies helped mitigate these impacts, disparities in vaccine uptake persisted due to complex social, behavioral, and structural factors (2, 5). For instance, in Latin America, socioeconomic challenges and low education levels hindered vaccine acceptance (6), while globally, misinformation and weak public health strategies fueled vaccine hesitancy (7). Multi-faceted interventions, such as social mobilization and targeted communication training for healthcare workers, demonstrated potential but required adaptation to specific community needs (8).

Kenya was no exception to these challenges. The country experienced significant impacts from the COVID-19 pandemic, with reported high transmission rates that led to over 0.34 million cases and a death toll exceeding 5,668 (9). However, there is no data on the country's COVID-19 vaccination uptake among persons with disabilities (PWDs) in Kenya despite their unique vulnerabilities, including chronic conditions and higher risk for severe outcomes. Through a national COVID-19 vaccine deployment plan, the government of Kenya prioritized vaccination as a key measure to contain COVID-19 spread that also targeted population groups, including PWDs scheduled to be vaccinated in phases (10). However, disaggregated data on proportions of vaccinated PWDs has been conspicuously missing on the COVID-19 vaccination Ministry of Health (MoH) update reports, making it difficult to track the progress made in reaching this cohort (9). Moreover, despite the availability of several studies on drivers of vaccination among other key population groups (11), there is dismal to no evidence of this among PWDs in low—and middle-income countries (LMICs).

Approximately 37% of Kenyan adults and 10% of children between ages 12 and 18 had been fully vaccinated as of December 2022 (12). Willingness for vaccine uptake was reportedly lower among younger people compared to the older; students compared to those working; and in the Coastal and Northeastern regions but higher in Nairobi and Rift Valley regions. Moreover, vaccine confidence was significantly associated with vaccine uptake, with Kenyans with lower confidence in vaccine safety being more likely to refuse the vaccine (11). Such studies, however, fail to account for influences specific to PWDs, bearing in mind their unique vulnerabilities.

Inadequate information on health inequalities has been identified as a distinct gap in strengthening inclusion and equity in COVID-19 responses (13). The scanty available evidence globally shows lower COVID-19 vaccination rates compared to those without disability (14) amidst higher risk of severe illness and premature mortality from COVID-19 (15).

This study was therefore conducted to investigate uptake levels of COVID-19 vaccines and to determine the behavioral and social predictors of COVID-19 vaccine uptake among PWDs in Kenya. Predicated on previous studies, the study hypothesizes that having a disability, residing in a lesser advantaged area, being of older age, having a caregiver and/or assistive device, significant risk perception, vaccine confidence, and strong health worker recommendation have positive association with COVID-19 vaccine uptake, whereas gender, education, employment and religion do not. The information on levels and drivers of vaccine uptake among persons with disabilities will better inform national last mile COVID-19 vaccination programs, and access and inclusion strategies for people with disabilities in similar vaccine-preventable disease programs in the future. This study is grounded in the WHO's Behavioral and Social Drivers (BeSD) framework, which provides a robust foundation for examining the social, cognitive, and practical factors influencing vaccine uptake. Applying this framework allows for a systematic exploration of the variables that contribute to COVID-19 vaccination behavior among persons with disabilities, contextualizing our findings within broader public health research.

## Materials and methods

### Study design

We employed a convergent parallel mixed-methods design, conducting quantitative and qualitative components simultaneously within the same research phase. Both methods were given equal weight and analyzed independently.

### Theoretical framework

The study objectives and variables were premised on the WHO's Behavioral and Social Drivers (BeSD) framework. This comprehensive framework consists of four main domains: (i) the thinking and feeling domain, which encompasses individual risk and benefit perceptions; (ii) social processes, including social norms; (iii) motivation; and (iv) practical issues, such as vaccine availability, access factors, quality of service, and the reception by healthcare workers. The BeSD framework is designed to enable health programs to collect, analyze, and utilize data effectively to understand the factors influencing vaccine uptake, thereby informing planning and implementation strategies (16).

Our study incorporated elements from the BeSD framework by leveraging its standardized tools and guidance during the development of data collection instruments, including both questionnaires and qualitative interview guides. This approach facilitates comparisons with other COVID-19 vaccine uptake studies conducted globally under the same framework, promoting consistency and cross-contextual learning.

In this study, the BeSD framework was adapted to assess key variables likely to influence COVID-19 vaccine uptake among persons with disabilities. These variables were categorized into two main groups: socio-demographic factors and behavioral factors, which were considered as independent variables. The dependent variable was defined as the uptake of the COVID-19 vaccination.

This theoretical grounding provided a structured way to analyze how individual, social, and practical factors interact to influence the decision-making process regarding COVID-19 vaccination among PWDs. The framework's application enabled a comprehensive examination that aligns with global studies on vaccine uptake.

### Study setting

The study was conducted in four counties in Kenya, selected based on their disability prevalence and representing the main regions of the country to attain a representative rural-urban mix. The counties were Embu in Eastern Kenya, with the highest disability prevalence of 4.4%, Siaya (4.1%) in the Western side, Mombasa (1.4%) in the Coastal region, and Nairobi (1.1%) in the Central region.

### Participants

The study participants were PWDs in the hearing, communication, self-care, cognition, mobility, visual and albinism domains, as per the Washington Group on Disability Statistics (WG). The study participants were chosen from the National Council of People with Disabilities (NCPWDs) registration databases of Mombasa, Siaya, Embu and Nairobi Counties. Albinism was included as it is classified as a disability in Kenya because of the associated visual impairment and other vulnerabilities in society, including mistreatment and exclusion (17). All participants were 18 years and above. Critically ill PWDs and those who declined participation in the study were excluded.

### Study process, sampling and data collection

The study was conducted in March 2023, about 2 years after rolling out Kenya's national COVID-19 vaccination program. Both quantitative and qualitative data collection took place within a 5-day period from 27^th^ to 31^st^ March 2023. The data collection was structured for efficiency and accuracy, involving a team of 29 trained research assistants distributed across four counties based on sample size needs for each county. Each research assistant received in-depth training, and fieldwork was closely supervised by Amref staff who conducted spot checks on collected questionnaires and performed call-backs to verify respondents' participation. PWDs were systematically chosen using a K^th^ of four to reach a sample size of 792 from the National Council for PWDs Registration database within Mombasa, Siaya, Embu and Nairobi counties. The respondents were contacted prior to field activity, while those not found were replaced by choosing the next participant based on the skipping pattern of four. Qualitative data collection, including 7 FGDs and 4 KIIs, was conducted concurrently by an additional team. This qualitative phase was completed within 3 days and aligned with data saturation principles. Purposive sampling was used to select key informants for in-depth interviews at both the county and national levels for qualitative data. The same technique was used to select 6 to 12 persons with disabilities who participated in the focus group discussions and chosen to represent a diverse cross-section of persons with disabilities (PWDs) across domains such as self-care, mobility, hearing, and cognition.

**Figure 1:**
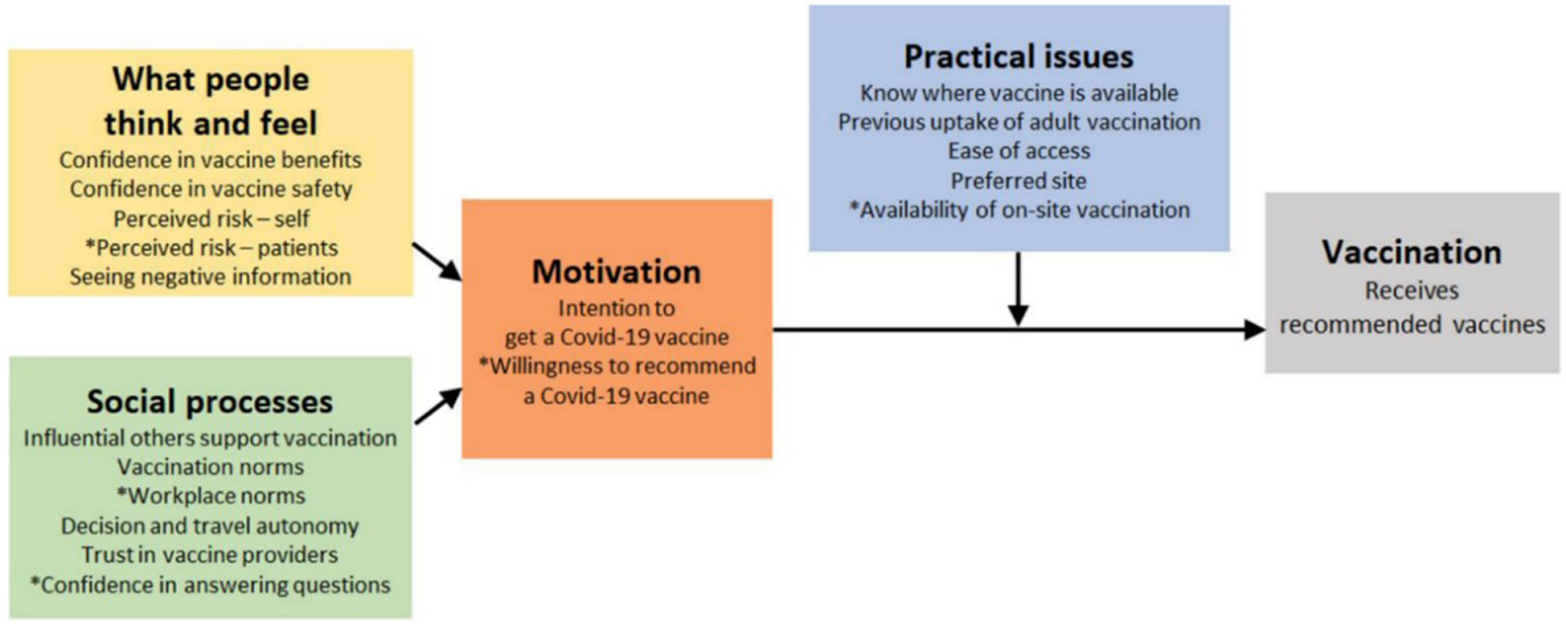
The Behavioral and social drivers (BeSD) framework. Source: Behavioral and social drivers of vaccination: tools and practical guidance for achieving high uptake. Geneva: World Health Organization and UNICEF; 2022.

A structured questionnaire embedded in a mobile collection tool (KOBO) was used to collect quantitative data from PWDs in the selected counties. Internal checks were done within the tool to ensure accuracy and completeness. Moreover, Focus Group Discussions (FGDs) were conducted among PWDs and their caregivers, while Key Informant Interviews (KIIs) were done among disability service officers, social development officers, COVID-19 vaccination in-charges and NCPWD representatives in the target counties. The WHO's framework on Behavioral and Social Drivers (BeSD) of vaccination, displayed in Figure 1 informed the data collection tools. Research assistants were trained on data collection methods, including consenting and interviewing processes, and on the various disability domains and related disability etiquette. Interviews were done in languages understandable to the respondents, with sign-language interpreters engaged to administer interviews to the hearing-impaired.

### Data collection instruments

In this study, qualitative data collection was carried out using semi-structured Focus Group Discussion (FGD) and Key Informant Interview (KII) guides. These guides were developed to capture a comprehensive understanding of the factors influencing COVID-19 vaccine uptake among persons with disabilities (PWDs), aligning with the WHO's Behavioral and Social Drivers (BeSD) framework. The FGD guide comprised approximately 11 key questions, focusing on areas such as perceived COVID-19 risk, vaccine confidence, social norms, and practical issues related to vaccine access. Each main question was supported by probes to facilitate a deeper exploration of participants' experiences and opinions. The KII guide, tailored for officials and community representatives, contained about 11 main questions covering topics like community perspectives, healthcare recommendations, and strategies to enhance vaccine access for PWDs. Probing questions were included to delve into aspects such as vaccine safety perceptions and challenges faced by PWDs during the pandemic. The semi-structured nature of these guides ensured consistency across interviews while allowing flexibility for participants to share detailed insights, enriching the data collected for thematic analysis.

Similarly, the survey questionnaire for this study was developed based on the BeSD framework. This framework provided a structured approach to capture key behavioral and social drivers across four main domains: thinking and feeling about vaccines, social processes influencing vaccination, motivation or hesitancy toward vaccination, and practical issues related to accessing vaccines. The questionnaire was organized into three main sections. Section A captured demographic information, including participant age, gender, marital status, education level, employment status, and disability status. Section B collected socio-economic data, with questions focusing on household composition, living arrangements, caregiving responsibilities, and the use of assistive devices. Section C addressed COVID-19 vaccine uptake, including questions on perceived COVID-19 risk, vaccination history, motivation for vaccination, sources of information, and practical challenges related to vaccine access. Each section was designed with detailed items allowing for both quantitative and qualitative responses, enabling a comprehensive assessment of beliefs, social influences, and access-related issues. The questionnaire was administered by trained interviewers who followed standardized instructions, including directives for optional questions and emphasis on priority items. This structured approach ensured consistent data collection, capturing distinct insights into vaccine-related attitudes and behaviors across diverse respondent demographics.

### Data management and analysis

All data collected through KOBO were first transferred to Excel for cleaning and later to STATA (version 14) for analysis. Descriptive statistics, including mean, standard deviation and frequencies, were generated. Tables and bar graphs were used to present the frequencies. Additionally, χ^2^ tests were used to test associations between behavioral and social predictors with the uptake of the COVID-19 vaccine. Cross-tabulation was used to describe data and to explain the relationship between COVID-19 uptake and the above independent variables. Multivariate regression was used to investigate how COVID-19 uptake was related to demographic, behavioral and social predictors, among other independent variables. The statistical significance level was set at *p* < 0.05 (18). For qualitative data, all interviews were transcribed and coded for analysis using NVivo software. Themes were generated using WHO's framework on the Behavioral and Social Drivers (BeSD) of vaccination, and deductive content analysis was used for analysis.

## Results

### Quantitative findings

#### Descriptive data

A total of 792 respondents were interviewed from four counties, namely Nairobi 321 (40.5%), Embu 188 (23.7%), Siaya 166 (21%), and Mombasa 117 (14.8%). Significant proportions of the PWDs who responded were urban, 350 (44.4%) and rural dwellers, 284 (36%), with about 19% residing in peri-urban areas. The mean age of the respondents was 44 ± 0.6 years, with more than half being male (56.2%) and a significant proportion being either married (47.6%) or single (41.8%).

The majority reported mobility impairments (62.8%), while those with albinism were the least (1.3%). There was an average of 4 persons in the households visited, with more than half indicating that they had a primary caregiver (52.9%) and used an assistive device (50.9%). Most (45.8%) had attained secondary education, while 35.4% had attained primary education. Slightly more than half (52%) were unemployed, with 31% engaged in self-employed activities. Moreover, over 90% were Christians, with a paltry being Muslims, as shown in Table 1.

**Table 1:** Socio-demographic characteristics of PWDs participating in the survey in 4 Kenyan counties.

**Variable name**	**Categories**	***n* (%)**
County	Embu	188 (23.7)
	Nairobi	321 (40.5)
	Siaya	166 (21)
	Mombasa	117 (14.8)
Residence	Rural	284 (35.9)
	Urban	350 (44.2)
	Peri-urban	158 (19.9)
Age of participant	Mean (SD)	44.0 (0.6)
Sex	Male	445 (56.2)
	Female	346 (43.7)
	Intersex	1 (0.1)
Marital status	Single	331 (41.8)
	Married	377 (47.6)
	Others (Divorced, separated or widowed)	84 (10.6)
Disability domain	Mobility	531 (62.8)
	Seeing	112 (14.4)
	Cognition	88 (11.0)
	Hearing	45 (5.7)
	Self-care	72 (9.2)
	Communication	52 (6.7)
	Albinism	10 (1.3)
Number of people in the household	Mean (SD)	4.5 (0.1)
Primary caregiver	Yes	419 (52.9)
	No	373 (47.1)
Use of assistive devices	Yes	403 (50.9)
	No	389 (49.1)
Level of Education	Madrasa	7 (0.9)
	None	98 (12.4)
	Primary	280 (35.4)
	Secondary	363 (45.8)
	Post-secondary	44 (5.5)
Employment Status	Employed	122 (15.4)
	Farming	11 (1.4)
	Retired	9 (1.1)
	Self employed	246 (31)
	Unemployed	412 (52)
	Christianity	724 (91.4)
	Islam	60 (7.6)
	Others (Jewish, atheists, and Buddhists)	8 (1.0)

#### COVID-19 vaccine uptake among persons with disabilities

About 59% (*n* = 469) of persons with disabilities interviewed reported to have been fully vaccinated against COVID-19, with 68% (*n* = 539) reporting to have received at least one dose, and 22% having received more than three doses.

The highest vaccinated by disability domains were those with albinism (70%), vision (66%) and mobility (61%) impairments, whereas individuals with cognitive (34.2%) and self-care (36.6%) were the least vaccinated.

#### Sociodemographic and behavioral factors influencing vaccine uptake

Chi-Square test of demographic factors showed that county, residence area, age and marital status were significantly associated with vaccine uptake. Additionally, education level, employment status, and religion were associated with vaccine uptake in the univariate analyses, as shown in Table 2. However, there was no significant relationship established between sex (Table 3) or history of COVID-19 diagnosis and vaccine uptake. Individual and societal behavioral factors that were shown to influence vaccine uptake were perceived risk to self, confidence in vaccine safety and vaccine benefits, family norms, religious leaders' norms, recommendation by health workers, recall notification, knowing where to get vaccinated, and ease of access. Community leaders' opinions did not significantly influence vaccine uptake. No association was also found between PWDs requiring permission to go for the vaccine and getting vaccinated.

**Table 2:** Social and behavioral factors influencing COVID-19 vaccination uptake among PWDs.

**Variable**	***n* (%)**	**Statistical significance**
Primary care giver		**0.003** ^*^
Yes	230(54.5)	
No	239(65.2)	
Use of assistance device		0.864
Yes	272(67.3)	
No	197(51.2)	
**Behavioral factors**		
Perceived risk-self	406(51.1)	**<0.001** ^*^
Confidence in COVID-19 vaccine benefits	460(64.1)	**0.04**
Confidence in vaccine safety	448(67.1)	**<0.03**
Confidence in health worker	752(95)	**<0.001** ^*^
Family norms	412(74.9)	**<0.001** ^*^
Religious leader norms	454(71.4)	**<0.001** ^*^
Community leader norms	431(69.4)	0.121
Health worker recommendation	374(88)	**<0.001** ^*^
Received recall	334(83.5)	**<0.001** ^*^
Knowledge on where to get vaccinated	522(96.8)	**<0.001** ^*^
Ease of access	510(59)	**<0.001** ^*^

^*^Bold indicates significantly associated results.

**Table 3:** Socio-Demographic factors influencing COVID-19 vaccination uptake among PWDs.

**Variable**	***n* (%)**	**Statistical significance**
County		**<0.0001** ^*^
Embu	321 (40.5)	
Nairobi	188 (23.7)	
Siaya	166 (21.0)	
Mombasa	117 (14.8)	
Area of residence		**0.004** ^*^
Rural	157 (55.7)	
Urban	235 (66.8)	
Peri-urban	77 (49.7)	
Age		**<0.001** ^*^
18–24	98 (13.4)	
25–34	101 (13.8)	
35–44	153 (20.9)	
45–54	174 (23.8)	
>64	94 (12.8)	
Sex		0.864
Male	261 (58.9)	
Female	207 (60)	
Intersex	1 (0.1)	
Marital status		**<0.001** ^*^
Single	165 (50)	
Married	255 (68)	
Separated	16 (50)	
Widowed	29 (64.4)	
Divorced	4 (57.1)	
**Education level**		**<0.001** ^*^
None	34(4.31)	
Madrassa	3(0.38)	
Primary	139 (17.6)	
Secondary	152(19.3)	
Post-Secondary	141 (17.9)	
**Employment status**		**<0.001** ^*^
Formally employed	102 (79.7)	
Self employed	167 (69.6)	
Unemployed	199(48.1)	
**Religion**		**0.032** ^*^
Christianity	437(60.9)	
Islam	29(46)	
Others	3(37.5)	

^*^Bold indicates significantly associated results.

The key motivations expressed by PWDs for getting vaccinated were to protect themselves (92.9%), protect their family (75.5%), and gain access to public spaces (30.6%). Low vaccine uptake was mainly attributed to perceived adverse vaccine effects (35.3%) and inadequate information (20.5%), with lack of information notably higher among persons with cognition impairments.

On social norms influencing vaccine uptake among PWDs, whereas PWDs believed that their close family & friends and religious leaders wanted them to get vaccinated, they trusted the healthcare workers' recommendation most (80%), and close family & friends second (39%). Other trusted social contacts were social service officers (14%), caregivers (12%), and religious leaders (8%). Despite high trust in health worker, the study found out that over 30% of PWDs had not been reached by health workers and the vaccine recommended to them. Moreover, four in ten PWDs reported low ease of access to vaccination services (*p* < 0.001), with the main reasons cited as difficulty getting to vaccination sites and long waiting times. Notably, lower ease of access was reported among persons with self-care (47.8%), cognition (48.7%), and vision (50%). A paltry (4%) were vaccinated door-to-door, despite home administration being the most recommended approach by PWDs for reaching them with vaccines.

Upon further analysis, the final logistic regression model showed age, county, employment status, perceived risk, confidence in vaccine benefits and health worker recommendation as the statistically significant socio-behavioral predictors of COVID-19 vaccine uptake among PWDs (Table 4). Older persons were more likely to get vaccinated compared to the younger age groups. For instance, persons aged 35–44 years were two times less likely to be vaccinated than those aged 64 years and above. PWDs in eastern counties were less likely to be vaccinated compared to those from the western region. According to this data, the employed were 2.63 times more likely to be vaccinated than the unemployed. Moreover, PWDs expressing confidence in COVID-19 vaccine benefits were 11 times more likely to take the vaccine, and those with high self-risk perception of the COVID-19 vaccine were twice less likely to take the jabs. Moreover, PWDs who had received health worker recommendations were 2.5 times more likely to take the jab, as shown in Table 4.

**Table 4:** Logistic regression on COVID-19 vaccination uptake predictors among PWDs.

**Variables**	**B**	**Sig**.	**OR**	**95% CI**
Age (Ref: > 64)		**<0.001** ^*^		
18–24	−1.717	**<0.001** ^*^	0.18	0.089–0.361
25–34	−0.714	**0.023** ^*^	0.49	0.265–0.906
35–44	−0.692	**0.033** ^*^	0.5	0.265–0.944
45–54	−0.437	0.171	0.646	0.345–1.208
55–64	−0.081	0.816	0.922	0.466–1.826
County (Ref: Siaya county)		**<0.001** ^*^		
Embu County	−0.733	**0.004**	0.481	0.291–0.793
Mombasa County	−0.212	0.457	0.809	0.463–1.414
Nairobi County	0.232	0.346	1.261	0.778–2.044
Employment Status (Ref: Unemployed)		**<0.001** ^*^		
Formally employed	0.968	**0.001**	2.63	1.51–4.57
Self employed	−1.64	0.145	0.195	0.022–1.76
Perceived risk - self	0.693	**0.001**	2.0	1.304–3.068
Confidence in COVID-19 vaccine benefits	2.421	**<0.001** ^*^	11.26	5.234–24.221
Health worker recommendation	0.936	**<0.001** ^*^	2.551	1.776–3.662

^*^Bold indicates significantly associated results.

### Qualitative findings

We derived a total of 23 unique codes from the qualitative data. Each code represented a specific concept or observation captured from participant responses, such as “Risk to self,” “Confidence in vaccine,” and “Family support/permission to vaccinate.” These codes were organized into broader categories to help identify patterns and connections within the data, with categories such as “Risk Perception,” “Social Influences,” and “Access and Practical Issues.” Finally, we synthesized these categories into overarching themes that provided insights into key findings. Qualitative findings revealed unique motivations for PWDs, for instance, getting vaccinated to safeguard oneself against additional exposure from assistive devices, e.g., white canes and reading braille. The influence of the political class on vaccine uptake was also cited in several accounts by PWDs on getting the vaccine upon seeing their political leaders, notably the president, getting it.

Perceived vaccine effects were also conspicuously reported among respondents in the qualitative findings, with a majority reporting having heard of the effects from external sources and hardly any from personal experience. Other notable reasons attributed to low uptake were distance and associated cost implications. One of the respondents recounted, “*The vaccination place was far from our residential area. Most of the people didn't get vaccinated because they didn't have transport money to move from the residential area to where the vaccination was taking place.”*—Visually Impaired FGD Respondent from Siakago, Embu County.

The qualitative findings similarly revealed that health workers were considered as credible sources of information, as recounted for instance: “*If I hear this information from a medical person, I would believe [it] because they have knowledge in the field. I would trust them [healthcare providers], but if I get it [information] from people around here I would not trust the vaccine since people have so many things going on. If it [information] comes from a religious leader, I will accept but not believe since that is not their area of specialty.”*—–A Physically Impaired FGD Respondent from Mbeere North, Embu County. Health workers were however required to provide clear information to influence vaccine uptake, as recounted “*If safety is guaranteed and the healthcare providers give clear [information] and sensitize, there will be an increase the uptake.”*—The Regional In-charge of the National Council for PWDs for Meru, Tharaka Nithi, Isiolo, and Marsabit counties. Key recommendations made were on working with PWD organizations and their social networks to reach people in all disability domains, intensifying sensitization of PWDs for informed decisions, giving special consideration to PWDs, equipping health workers to handle PWDs, and making transportation arrangements to vaccination sites for PWDs.

## Discussion

The proportion of PWD respondents who indicated being fully vaccinated was higher than the general population proportion vaccinated in Kenya (36.9%) during the study period. COVID-19 vaccination provides significant health benefits for persons with disabilities (PWDs), including reduced disease severity, shorter hospital stays, and lower mortality rates compared to unvaccinated individuals (19). However, barriers such as physical inaccessibility, limited transportation options, and difficulties in accessing information have contributed to lower vaccination rates among PWDs relative to the general population (20). These barriers place unvaccinated PWDs at higher risk of severe COVID-19 outcomes due to underlying health conditions and limited ability to adhere to preventive measures, which further heightens their vulnerability (21). Additionally, the pandemic exacerbated the existing inequalities faced by PWDs, impacting their access to healthcare, education, and employment opportunities (22). Addressing these disparities requires prioritizing PWDs in vaccination strategies and implementing targeted efforts to improve accessibility, such as reducing logistical barriers and enhancing the accessibility of vaccination sites (20, 21). This points to deliberate interventions to get PWDs vaccinated and higher acceptance levels.

However, those in the self-care and cognition domains were disproportionately vaccinated compared to other domains. A 2022 survey found that persons with cognitive or intellectual disabilities lacked easy-to-read, simpler text versions of public information and communication material, which made decision-making difficult (23). Another study among people with intellectual and developmental disabilities and their families showed that COVID-19 vaccine uptake was associated with self-reported knowledge about the vaccine and learning about the vaccine from one's doctor, among other variables (24). Accessible education and support from healthcare providers and caregivers are significant in addressing these disparities (25).

Regarding the low vaccine uptake among young PWDs, Osur et al. established the main causes of vaccine hesitancy among youth in Kenya as concerns about vaccine safety and effectiveness (26), which corroborates the findings of our study. Higher vaccine hesitancy was also reported in younger persons with intellectual and developmental disabilities in New York state individuals by Iadarola et al. (27). Some studies have also reported a lower likelihood of vaccine hesitancy among older adults (28, 29).

Past related studies in Kenya have also shown low confidence in COVID-19 vaccines in the coast region (67%), Nyanza (76%), and Eastern (78%) compared to Nairobi (79%). This aligns with our findings of significantly low vaccine uptake in Mombasa in the coastal region. However, a study by Orangi et al. on determinants of vaccine confidence in Kenya presents contradicting results, indicating that rural counties had higher odds of reporting vaccine hesitancy (aOR = 2.46; 95% CI: 1.02–5.94) compared to urban counties like Mombasa (30).

Unlike many studies that show no association between employment and vaccine uptake among other population cohorts, our study found a significant association. The requirement for government employees to be vaccinated may explain the higher vaccination rates among employed PWDs, while transportation costs and other logistical challenges may have hindered uptake among unemployed PWDs. Decentralizing vaccination services for PWDs and providing free transportation to vaccination facilities may increase vaccine uptake rates among the unemployed PWDs.

The strong influence of high vaccine confidence and risk perception is corroborated in prior studies (31–34), with the pandemic reported to have had a positive impact on general vaccine confidence in Kenya. Masters et al. *also cited low confidence in the* COVID-19 vaccine as the strongest correlate of not taking COVID-19 vaccines (35). Orangi et al. also linked risk perception to vaccine confidence with those perceiving COVID-19 as not risky being likely not to get vaccinated (30). However, in a study by Alobaidi perceived severity was not significant in predicting vaccine uptake intentions (36). Vaccination communication campaigns and advocates should inevitably consider inspiring vaccine confidence. The “Stop HPV, Stop Cervical Cancer” by the Danish Health Authority, the Danish Cancer Society, and the Danish Medical Association is a classical campaign building vaccine confidence (37). The study established the key motivations of protecting oneself and significant others, which could be capitalized on alongside the messages raising vaccine confidence. However, when communicating to PWDs, the motivations should be contextualized to include routes of transmission unique to this cohort, including possible infection through assistive devices and caregivers.

The major impediment to vaccine uptake was perceived vaccine effects (26, 30, 33, 36, 38). In a study among Americans with disabilities, vaccine effects were similarly glaring, with the study highlighting higher concerns about COVID-19 vaccine safety as compared to concerns about contracting the disease.

Healthcare providers and family members have been similarly cited as trusted sources of information by PWDs (27). Latkin et al. also found out that close family and friends discouraging vaccination was a key predictor of low vaccine uptake (aOR = 0.26, 95% CI = 0.07–0.98) (39). Vaccination programs and influencers, including social services officers and religious leaders, should, therefore, not only reach PWDs but also their close social contacts, considering their influence. The high trust in health workers has been consistently attributed (40–43) with the information they provide linked to better health access (44). With vaccine hesitancy reported even among health workers (45), despite perceived COVID-19 severity, prevention and vaccine safety, a study by Iliyasu et al. recommends a multidimensional approach to increasing vaccine acceptability (46). Many healthcare professionals, however, lack information and may feel hesitant to provide accurate responses (47) which necessitates capacity building of health workers and other influencers of persons with disabilities to adequately address patients' questions and concerns regarding vaccination. Communication on vaccine uptake should be grounded in the key principles of science-based evidence and data, transparency (i.e., acknowledging to the public what is not yet known), and communicating clearly to achieve understanding by all persons, as recommended by NIH's Dr. Anthony Fauci (48). This would help bridge perceived negative effects of vaccines and lack of information that were mainly linked to vaccine hesitancy.

The low vaccine uptake attributed to low ease of access has been shown to be bridged by localizing vaccination services, reducing the difficulty of getting to vaccination centers. A study found that a community-based health effort utilizing a mobile vaccination clinic successfully increased COVID-19 vaccine adherence among the Black population in San Bernardino County with observed lower uptake rates (49). Prioritizing vulnerable populations and centralized fixed-time appointments for receiving the vaccines could potentially save them from the long wait. Working with PWDs' organizations and networks, focusing awareness efforts, customized accommodations, and specialized training for healthcare professionals have also been recommended to improve vaccine uptake among this cohort.

### Implications

This study highlights several key areas for improving vaccine uptake among persons with disabilities (PWDs). Tailored communication strategies, including accessible formats and clear messaging, are essential to address vaccine hesitancy. Engaging healthcare providers, caregivers, and family members is also crucial, as they serve as trusted sources of information. Policies should prioritize decentralized vaccination services, transport support, and ensuring PWDs are prioritized in vaccine distribution. Community-based initiatives, such as mobile clinics, and fixed-time appointments can further enhance access.

Clinically, healthcare providers should be trained in disability-inclusive care, focusing on clear communication and addressing vaccine concerns. Involving family members and caregivers in the vaccination decision-making process is also vital. Workplace vaccination policies, such as paid leave and transport assistance, could increase uptake among employed PWDs, and extending these policies to rural and smaller employers may further improve access.

From a policy perspective, disability-inclusive health strategies should be integrated into national vaccination campaigns. Collaborating with disability advocacy groups to design accessible and supportive interventions will help ensure that PWDs are included in future vaccination efforts.

### Limitations

While this study provides valuable insights into vaccine uptake among PWDs, a few limitations should be considered. First, the study's cross-sectional design limits the ability to infer causal relationships between the identified factors and vaccine uptake. Additionally, the sample was limited to specific regions and may not be fully representative of the broader PWD population in Kenya, particularly those in rural or hard-to-reach areas. Although efforts were made to ensure diverse participation, there may be regional variations in attitudes toward vaccination that were not fully captured in this study. The reliance on self-reported data also introduces the possibility of response bias, which is common in survey-based research, as participants may have over-reported vaccination status or may have been influenced by social desirability bias. Finally, while we focused on understanding the perspectives of PWDs, further research that includes the views of healthcare providers and policymakers would provide a more comprehensive understanding of the barriers and enablers to vaccine uptake from a broader, systemic viewpoint.

## Conclusion

In conclusion, this study highlights the uneven COVID-19 vaccine uptake across disability domains, with significantly lower rates among PWDs in cognition impairment and self-care domains. According to the findings, confidence in vaccine benefits, being employed, perceived self-risk and health worker recommendation were associated with high vaccine uptake. In contrast, perceived effects were attributed to low uptake. The influence of health worker recommendations on vaccination choices highlights the significance of healthcare provider participation in vaccine outreach. Improving immunization rates among PWDs requires addressing issues with access, vaccination site difficulties, and long waiting times. Targeted tactics, including collaborating with disability organizations, raising awareness among PWDs, and setting up transportation, can be implemented to guarantee that everyone has fair access to vaccinations.

## Data Availability

The raw data supporting the conclusions of this article will be made available by the authors, without undue reservation.
